# Serum Nerve Growth Factor Levels as a Predictor of Bull Candidate Semen Quality of Madura Cattle

**DOI:** 10.1155/2022/7128384

**Published:** 2022-04-11

**Authors:** Rimayanti Rimayanti, Nurul Azizah, Pudji Srianto, Sri Pantja Madyawati, Trilas Sardjito, Imam Mustofa

**Affiliations:** ^1^Faculty of Veterinary Medicine, Airlangga University, Kampus C Mulyorejo, Surabaya 601155, East Java, Indonesia; ^2^Indonesian Research Institute for Animal Production, Indonesian Agency for Agricultural Research and Development, Jalan Veteran III, Ciawi, Bogor, Indonesia

## Abstract

Madura cattle are the germplasm of native cattle on the verge of extinction because of crossbreeding. Therefore, the present study was aimed to determine the serum nerve growth factor (NGF) concentration as a predictor of fresh ejaculate fertility parameters in Madura bull candidates. Eleven Madura bull candidates used for frozen semen production were selected for the study. Blood samples were collected using a vacutainer from the jugular vein for analyzing serum NGF and testosterone levels. Meanwhile, semen collection was conducted using an artificial vagina for sperm motility, viability, and concentration assessment. Data were analyzed to determine the correlation among variables and the linear regression of NGF concentration to other significant variables. The result showed that NGF had a significant correlation (*p* < 0.05) with testosterone levels, sperm motility, viability, and concentration. A significant correlation was observed between testosterone levels, sperm concentration, and sperm viability. The regression equation among significantly correlated variables was determined. For artificial insemination, suitable bull ejaculates for obtaining frozen semen should reach at least 2.12 ng/mL of NGF levels, with sperm viability, sperm concentration, and testosterone levels of more than 78.63%, 1,462.177 million/mL ejaculate, and 25.67 ng/mL, respectively. This is the first study to identify NGF as a predictor of male fertility in bull candidates of Madura cattle. Therefore, NGF levels could be used as a marker of male fertility in Madura bull cattle candidates. Thus, based on the minimum NGF levels, the ejaculate of Madura bull candidate that meets the requirements for frozen semen production could predict fertility.

## 1. Introduction

Madura cattle are germplasm of hybrid beef cattle native to Indonesia from crosses between Banteng (*Bos javanicus*) and Zebu cattle (*Bos indicus*). Farmers rear Madura cattle for savings, additional income, financial empowerment, manure production, depicting social status, and cultural value [1]. As the name implies, Madura cattle have been reared for centuries by the people of Madura Island, Indonesia. However, in the last decades, the farmers have preferred to crossbreed small local cows through artificial insemination techniques using the frozen semen of European beef breeds, such as Simmental and Limousin [2]. Meanwhile, there has been an annual increase in the demand trend [3]. Our field observation indicated that Madura cows inseminated with Simmental or Limousin post thawed frozen semen bull resulted in Metal and Madrasin cattle, respectively, as the local people call them. Indeed, crossbred beef cattle had higher meat production and higher market price [4] because of their characteristic heavy weight at birth and maturity; also, weaning calves have a higher body weight than native cattle [5]. Limousin crossbreed had good weight gain compared with Madura cattle [6]. Crossbreeding holds numerous economic benefits for farmers and ultimately increases national production [7]. However, crossbreeding between exotic and locally adapted cattle breeds can have harmful consequences and cause extinction [8].

The government of the Republic of Indonesia was concerned about the designation of Madura cattle as Indonesia's local cattle [9]. Thus, they issued a quality standard for Madura cattle breeds to improve the genetic quality and protect the genetic resources of local Madura cattle [10]. Sapudi Island, an isolated island in East Java Province, is the center for developing purebred Madura cattle. Reportedly, other cattle breeds cannot be reared on the island. Therefore, genetic quality must be increased following Madura cattle breeding through artificial insemination. Additionally, improving bull reproductive performance is necessary to optimize cattle production efficiency. Bull fertility has been genetically correlated with traits, such as average daily gain, pregnancy rate, and calving interval [11], which are regulated by growth factors [12]. A growth factor that affects the growth and development of the reproductive system is the nerve growth factor (NGF), which affects the growth of Leydig cells responsible for testosterone synthesis [13]. Testosterone plays a vital role in dividing germ cells to form spermatozoa, especially during meiosis. This process also leads to the formation of secondary spermatocytes [14]. However, to our knowledge, no known molecular marker standards have been established as a predictor of fertility selection in Madura bull candidates.

Therefore, this study aimed to determine the serum NGF concentration as a predictor of fertility in Madura bull candidates on the basis of serum testosterone concentration, sperm motility, viability, and concentration of fresh ejaculates as fertility parameters.

## 2. Materials and Methods

Semen quality assessment was conducted at the Pamekasan Animal Husbandry Service Laboratory, Madura. Meanwhile, serum NGF and testosterone levels were measured using enzyme-linked immunosorbent assay (ELISA) at the Institute of Tropical Disease, Airlangga University. This study protocol was examined and approved by the Animal Care and Use of Faculty of Veterinary Medicine, Airlangga University, with the reference number 792-KE.

### 2.1. Animal Management

In this study, 11 Madura bull candidates were used for frozen semen production. All bulls were aged 3–5 years, weighing 224–330 kg, fed elephant grass at 10% body weight and 9 kg of concentrate (16%–17% crude protein), and provided drinking water *ad libitum*. The bulls were reared at the Technical Implementation Unit, an animal facility for Livestock Breeding and Animal Health in Pamekasan Regency, Madura, East Java, Indonesia. Pamekasan regency is located 8 m above sea level at latitude 6°51′–7°31′L and longitude 113°19′–113°58′E. Pamekasan regency climate is cloudy in the wet season; however, it is windy and partly cloudy in the dry season. It is hot and oppressive year-round, with the temperature varying between 28°C and 35°C. The relative humidity is approximately 80%, and rainfall occurs six rainy days per month [15].

### 2.2. Samples

Blood samples were collected using a vacutainer from the Jugular vein in six replicate at the same time of semen collection. Blood samples were left in room temperature (24°C) until clot, then centrifuged with a speed of 10 xg for 10 minutes, pipetted the supernatant into cone tube and freeze-stored at −20°C. Semen collection of each bull candidate was conducted using an artificial vagina twice a week (Tuesday and Friday) for three weeks. Semen samples were immediately evaluated for sperm motility, viability, and concentration parameters.

### 2.3. NGF Measurement

Examination of NGF levels was conducted using indirect ELISA based on Kit protocol. The chemicals used were standard solution, anti-NGF antibody, streptavidin-HRP, and substrate solution antibody (all from Thermo Fisher Scientific, Melaka, Malaysia). The optical density value was measured using an ELISA plate reader at 450 nm wavelength. NGF concentration was obtained on the basis of optical density values of the standard and the samples using ELISA v.2.15 (CDC, NY, USA) [16].

### 2.4. Testosterone Measurement

Measurement of testosterone levels was conducted using indirect ELISA based on Kit protocol. The chemicals used were testosterone antigen solution (Abcam, Cambridge, UK), buffer solution (Invitrogen, Melaka, Malaysia), blocking solution (Thermo Fisher Scientific, Melaka, Malaysia) testosterone antibody (Abcam, Cambridge, UK), and enzyme-conjugated secondary antibody (Abcam, Cambridge, UK). The optical density value was measured using an ELISA reader at 450 nm wavelength. Testosterone concentration was obtained on the basis of optical density values of the standard and the samples using ELISA v.2.15 (CDC, NY, USA) [17].

### 2.5. Assessment of Sperm Quality

The sperm concentration of Madura candidate bull semen was determined using a standardized spectrophotometer (Bovine Accucell photometer, IMV, L'Aigle, France). First, the sperm concentration was measured at 535 nm wavelength using a prewarmed and calibrated photometer. The photometer was set at zero using normal saline water, and then, 20 *μ*L semen samples were mixed in 2 mL of 0.9% w/v NaCl solution [18].

Six replications of each bull ejaculate sample were evaluated for sperm concentration, motility, and viability. Sperm viability assessment was conducted using a microscopic slide containing a drop of semen sample mixed homogeneously with a drop of eosin nigrosine (eosin 1%: nigrosin 5% in 1 : 1). This mixture was smeared and dried over a flame. The slide was examined at 400× magnification for 100 sperm under a light microscope (Olympus BX-53). The heads of live sperm appeared brightly transparent, whereas dead sperm were pinkish. The percentage of live and morphologically (head, neck, and tail) abnormal sperms was assessed for 100 sperm at 400× magnification [6]. Sperm motility was evaluated using a homogenized mixture of 10 *μ*L of semen and 10 *μ*L of physiologic saline (0.9% NaCl). Then, it was dropped on an object glass and covered. For every 100 sperm, the number of sperm showing progressive motility was counted using a 400× magnification microscope (Olympus BX-53) on a heating table at 37°C–38°C [6].

### 2.6. Data Analysis

Data of concentration of NGF, testosterone, sperm concentration, motility, and viability were tabulated and presented descriptively. Additionally, the normality was measured using the Kolmogorov–Smirnoff test. The correlation among variables was determined using the Pearson correlation coefficient. Furthermore, the linear regression of NGF concentration to the other significant variables was evaluated. A linear regression line has the equation: Y = a + b *∗* X, where “X” is the explanatory or independent variable and “Y” is the dependent variable. The slope (beta coefficient) of the straight line is “b,” whereas “a” is the intercept (the value of y when x = 0). The value of coefficient correlation (*r*) obtained from the analysis, i.e., 0.00–0.19, indicated a weak relationship among the variables. However, other studies implied that 0.20–0.39 is weak, 0.40–0.59 is moderate, 0.60–0.79 is strong, and 0.80–1.0 is a very strong relationship among the variables. The coefficient of determination, *R*^2^, represents the proportion (%) of the variance for a dependent variable that can be elucidated by an independent variable [19]. Statistical analysis was conducted at a 95% significance level using the Statistical Product and Service Solutions (SPSS, v.21; IBM Corp., Armonk, NY, USA).

## 3. Results

The data of NGF and testosterone levels, sperm motility, viability, and concentration were homogenous (*p* > 0.05) based on the Kolmogorov–Smirnoff test (Table 1). Pearson matrix correlation analysis indicated a significant correlation (*p* < 0.05) between NGF levels and other variables. The variables include testosterone levels (strong correlation), sperm motility, viability, and concentration in moderate correlation. Also, a strong correlation (*p* < 0.05) was identified between testosterone levels, sperm concentration, and sperm viability. The results also showed a robust correlation between sperm viability and motility. Meanwhile, there was no significant (*p* > 0.05) correlation between testosterone levels and sperm motility, and between sperm concentration, viability, and motility (Tables 2 and 3).

**Figure 1:**
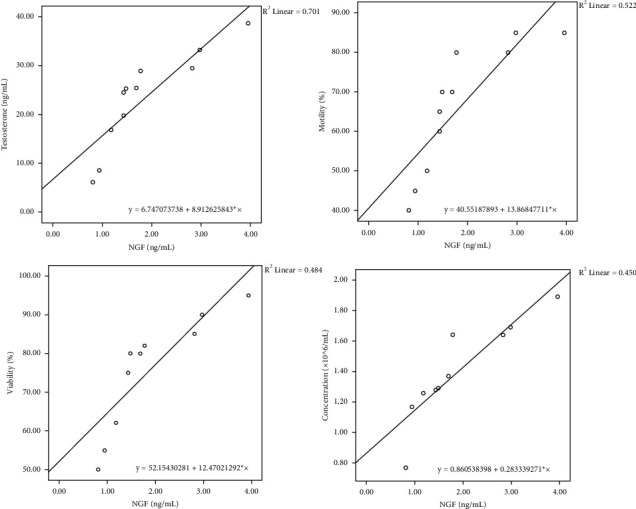
Scatterplot of serum testosterone levels, sperm motility, viability, and sperm concentration based on NGF levels as a predictor.

**Figure 2:**
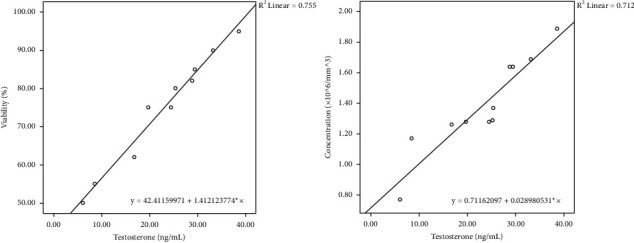
Scatterplot of sperm viability and sperm concentration based on serum testosterone levels as a predictor.

**Figure 3:**
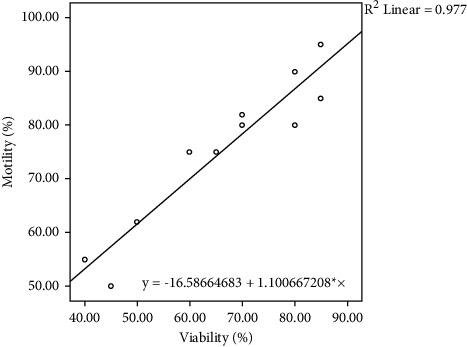
Scatterplot of sperm motility based on sperm viability as a predictor.

Table 4 and Figures 1–3 present the regression equation among significantly correlated variables. Bull ejaculates were suitable for frozen semen production, possessing at least 70% sperm motility, needed for artificial insemination [20]. Based on the regression equation, Mot = 40.55 + 13.87 *∗* NGF (regression equation no. 4, Table 4), the ejaculate of Madura candidate bull that meets the minimum requirements was obtained at NGF levels greater than 2.12 ng/mL. Furthermore, based on the NGF level at a minimum of 2.12 ng/mL, the sperm viability (regression equation no. 3, Table 4) must exceed 78.63%. Also, the sperm concentration (regression equation no. 2, Table 4) must exceed 1,462.177 million/mL ejaculate. Serum testosterone levels (regression equation no. 1, Table 4) should be greater than 25.67 ng/mL. Furthermore, a linear correlation was observed between testosterone levels, sperm concentration, and sperm viability and between sperm viability and motility (*p* < 0.05).

## 4. Discussion

This study selected 11 male Madura cattle as bull candidates for frozen semen production on the basis of age and morphometric performance criteria. Their NGF levels were evaluated and within the range of 0.808–3.946, with an average of 1.86 ± 0.29 ng/mL. NGF is a neurotrophin family with approximately 13 kDa molecular weight, which functions as a gonadotropin-releasing hormone (GnRH) agonist in cattle [21]. Additionally, NGF is produced in bulls' ampulla and vesicular glands [22] and is abundant within the vascular endothelium [23].Its concentration in the seminal plasma and blood plasma remains constant [24]. NGF and its receptor have also been identified in mice-ejaculated spermatozoa and human and bovine species. NGF plays a role in sperm viability and fertilizing ability [25]. Several studies have been conducted to improve male fertility in animals or humans using NGF. Furthermore, adding NGF promotes sperm motility and viability in bulls [26]. However, Sanchez–Rodriguez et al. [27] reported that *in vitro* addition of NGF to the ejaculate did not affect sperm viability, although the sperm motility parameters were enhanced. Human NGF promotes motility and vigor while maintaining viability and mitochondrial activity [28]. Also, supplementing freezing extender with NGF minimally affected post thawed sperm quality in bull [29] and human sperm [30, 31]. In females, NGF could act directly on theca and granulosa cells of the bovine preovulatory follicle to stimulate testosterone production, which may be secondary to theca cell proliferation [29].

The semen quality parameters of the Madura cattle bull candidate, including sperm motility, viability, and concentration, have been measured. The average sperm concentration (1,275.36 ± 309.76 million/mL) and motility (66.36 ± 15.98%) were within the ranges of 1,059 ± 421.5 million/mL to 61.6% ± 16.5% [32], and 0.96–1.13 million/mL to 61.3%–66.1% [33], respectively. They were higher than 1,050 million/mL and 41.62% ± 2.86% [34]. Furthermore, the semen quality parameters were correlated with NGF levels. Based on the coefficient of determination, data obtained that NGF affects the values of testosterone levels, sperm concentration, viability, and motility by 49, 20, 23, and 27%, respectively. Testosterone levels affect the values of sperm concentration and viability by 50 and 56%, respectively. Whereas, sperm viability affects the value of motility by 88%. The regression equation found can be used to predict the semen quality of bulls for Madura cattle bulls. These facts follow the existing theory as explained below.

The use of NGF levels as a predictor of semen quality in cattle has no reports found. The publications found were NGF addition on Llama (*Lama glama*) sperm traits after cooling [28] and the effect of NGF on the normozoospermic [30] and asthenozoospermic [31] men during the cryopreservation process. NGF plays an essential role in regulating the hypothalamus-pituitary-gonadal axis. Studies have shown that NGF upregulates markers expressing meiotic spermatogonia and spermatocytes, and improves sperm quality. Additionally, NGF mediates increased secretion of the follicle-stimulating hormone (FSH), luteinizing hormone (LH), and testosterone [35]. Normal spermatogenesis depends on specific signaling from the hypothalamic-pituitary-gonadal axis. GnRH, FSH, and LH are the primary hormones involved in spermatozoa production and maturation of [36]. Sperm motility is a vital parameter of spermatozoa fertilizing ability. Motility is regulated by several hormones and growth factors, such as testosterone, epidermal growth factor, and fibroblast growth factor [30, 31].

NGF is also secreted into the ejaculate and positively associated with bull fertility [22]. The role of NGF in male fertility is mediated through its binding to receptors responsible for autocrine and paracrine regulation of spermatogenesis. It promotes sperm motility, acrosome reaction, necrosis, and apoptosis [37]. Furthermore, NGF prevents premature sperm hyperactivation in ejaculated spermatozoa [29]. Also, it enhances sexual function, improves the quality of sperm, and restores fertility hypogonadism in male mice by activating GnRH [38]. In cattle, NGF enhances steroidogenesis through increased release of LH and downstream effect of increased interferon-stimulated gene expression. The positive association of NGF with bull fertility corroborates that NGF might be a tool to improve fertility in cattle [39].

Testosterone levels of Madura cattle bull candidates have been evaluated. They were within the range of 6.09 to 38.64, with an average of 23.34 ± 9.91 ng/mL. Statistical analysis indicated a positive correlation between NGF and testosterone levels. Furthermore, testosterone levels were also positively correlated with sperm concentration and viability. This results was confirming the hypothesis of Li and Zhou [40] that NGF may intervene in testosterone mediated spermatogenesis. Sperm production starts from puberty in the seminiferous tubules. Testosterone is produced by the Leydig cells in the interstice of the testicles [36]. Leydig cells can synthesize androgen (mainly testosterone) from cholesterol, and testosterone is transported by androgen-binding protein to Sertoli cells, which binds to the androgen receptor to regulate spermatogenesis [41]. Testosterone regulates spermatogenesis, which initiates the control of gonocyte numbers and spermatogonia expansion. This process also controls the completion of meiosis and attachment and ultimately releases elongated spermatids [42]. In this study, testosterone levels of male Madura cattle bull candidates were positively correlated with sperm concentration and viability. There is a positive correlation between serum testosterone concentration and fertility of bulls based on higher sperm motility, mass movement, concentration, and lower total defects in sperm [43].

Furthermore, this is the first study to determine NGF as a predictor of male fertility in Madura cattle bull candidates based on the equations mentioned above. The results indicated that NGF could be used as a marker of male fertility in Madura cattle bull candidates. The bull ejaculates, possessing at least 70% sperm motility, were suitable for frozen semen production needed for artificial insemination [20]. Using this standard, the mathematical regression equation of sperm motility variables could determine the NGF levels. The ejaculate of Madura candidate bull that meets the minimum requirements for frozen sperm production was obtained at NGF levels of at least 2.12 ng/mL.

Furthermore, based on the minimum 2.12 ng/mL NGF levels, other variables, such as sperm viability, concentration, and testosterone, must exceed 78.63%, 1,462.177 million/mL ejaculate, and 25.67 ng/mL, respectively. Therefore, the selection of male Madura cattle as bull candidates is recommended based on these minimum values. Also, based on the national standard qualification, the post thawed bull frozen semen must reach a minimum of 40% [20]. However, this study was limited to fresh semen quality parameters of Madura candidate bull. Therefore, further studies are needed to evaluate the post thawed semen quality and pregnancy rate of cows inseminated with straws.

## 5. Conclusion

The semen quality parameters (sperm motility, viability, and concentration), serum NGF, and testosterone levels were evaluated in male Madura cattle bull candidates. Results obtained indicated that NGF levels could be used as a marker of male fertility in Madura cattle bull candidates. Therefore, based on the minimum NGF levels, the ejaculate that meets the minimum requirements for frozen semen production could predict fertility in Madura bull candidates.

## Figures and Tables

**Table 1:** Serum nerve growth factor levels (ng/ml), serum testosterone levels (ng/ml), sperm motility (%), sperm viability(%), and sperm concentration (million/mL) of Madura cattle (n = 11).

Parameters	Min	Max	Mean ± SD	*p* value
NGF levels	0.808	3.946	1.86 ± 0.29	0.995
Testosterone	6.09	38.64	23.34 ± 9.91	0.850
Sperm motility	40.00	90.00	66.36 ± 15.98	0.411
Sperm viability	50.00	96.00	75.36 ± 14.20	0.635
Sperm concentration	361.00	1892.00	1275.36 ± 309.76	0.901

Min: minimum value; Max: maximum value; SD: standard deviation; *p* value: significance of probability.

**Table 2:** Pearson matrix correlation among variables (serum NGF levels, testosterone levels, sperm motility, sperm viability, and sperm concentration).

Parameters	NGF levels	Testosterone levels	Sperm Concentration	Sperm Viability
Testosterone levels	0.703^*∗*^			
Sperm concentration	0.450^*∗*^	0.712^*∗*^		
Sperm viability	0.484^*∗*^	0.755^*∗*^	0.232	
Sperm motility	0.522^*∗*^	0.555	0.199	0.94^*∗*^

^*∗*^Significant correlation (*p* < 0.05).

**Table 3:** Level significance of correlation coefficient (*r*) among variables (NGF levels, testosterone levels, sperm motility, sperm viability, and sperm concentration).

Parameters	NGF levels	Testosterone levels	Sperm concentration	Sperm viability
Testosterone levels	0.016			
Sperm concentration	0.041	0.014		
Sperm viability	0.026	0.007	0.492	
Sperm motility	0.015	0.076	0.557	0.00002

**Table 4:** Regression equation among significantly correlated variables.

No	Predictor	Parameters	Regression equation	*r*	*R* ^2^	*p* value
1	NGF levels	Testosterone levels	Tes = 6.75 + 8.91^*∗*^NGF	0.70	0.49	0.0004
2		Sperm concentration	Con = 0.86 + 0.28^*∗*^NGF	0.45	0.20	0.0002
3		Sperm Viability	Via = 52.15 + 12.47^*∗*^NGF	0.48	0.23	0.0008
4		Sperm motility	Mot = 40.55 + 13.87^*∗*^NGF	0.52	0.27	0.001
5	Testosterone levels	Sperm concentration	Con = 0.71 + 0.03^*∗*^Tes	0.71	0.50	0.00003
6		Sperm viability	Via = 41.41 + 1.41^*∗*^Tes	0.75	0.56	0.00003
7	Sperm viability	Sperm motility	Mot = −16.59 + 1.10^*∗*^Via	0.94	0.88	0.00002

*R*: coefficient of determination; r: coefficient correlation; *p*: significance of probability; NGF: nerve growth factor; Tes: testosterone; Con: sperm concentration; Via: sperm viability; Mot: sperm motility; *r*: correlation coefficient; *R*^2^: determinant coefficient.

## Data Availability

The data used to support the findings of this study will be made available on request.
